# Predictors of mortality in patients from a hematological ICU in Brazil

**DOI:** 10.1186/cc11015

**Published:** 2012-03-20

**Authors:** OB Silva, L Correa, P Loureiro, E Araujo, D Teles, LA Vasconcelos, T Salvattori, P Schwambach, GT Henriques-Filho

**Affiliations:** 1 HEMOPE, Recife, Brazil

## Introduction

The study was designed to analyze the factors responsible for increased mortality in an ICU specialized in hematological patients. There are few ICUs specialized in hematological diseases, with reports of high mortality rates (45 to 85%) [1], mostly related to severity of patients with blood cancer [2], mechanical ventilation (MV) and multiple organ failure [2-4]. The most prevalent disease differs among studies [1-4] and acute leukemia seems to have the worst prognosis [2].

## Methods

A retrospective cohort was conducted at HEMOPE's ICU. Data were collected from the medical records of patients admitted from January 2006 to December 2009.

## Results

Of the 576 admissions, 396 (68.75%) could be analyzed. The average age was 48.3 ± 19.4 years (11 to 88 years), 54% were female and there was no association between mortality and age or gender. Acute leukemia occurred in 43% (65.3% acute myeloid leukemia). Sepsis was the major cause of admission (55.3%). The overall mortality rate was 57.5% and the specific one was 42.7%. The mean APACHE II score for this population was 13.4 ± 1.0 (7 to 43) and was statistically higher in the group that died (14.6 ± 0.7 vs. 11.8 ± 0.8; *P *= 0.013). Mean SOFA at day 1 (D1) and day 3 (D3) was 2.8 ± 0.2 and 2.1 ± 0.2 respectively, also significantly higher in those that died (D1 3.9 ± 0.3 and D3 2.9 ± 0.3; *P *< 0.0001). Almost 60% used vasoactive drugs (VAD) on admission and had a higher mortality rate (*P *< 0.0001). MV was used in 86% and 69% died (*P *< 0.001). Of those with renal substitutive therapy (RST), 81.9% died (OR = 3.12; 99% CI = 1.5 to 6.91). Mortality was also associated with the completion of chemotherapy before ICU admission (*P *= 0.003) and severe neutropenia (*P *< 0.0001). In multivariate analysis, MV (RR = 13.1; 99% CI = 5.14 to 33.45) and a one-unit increase in SOFA D1 (RR = 1.26; 99% CI = 1.15 to 1.37) were associated with an increase in mortality.

## Conclusion

For this population, in univariate analysis mortality was related to SOFA, RST, MV, use of VAD on admission, chemotherapy before ICU admission, and severe neutropenia. Although there was a relation between APACHE II score and mortality, this score underestimates it. In multivariate analysis, needing MV and a high SOFA D1 were independent predictors of death.
